# The longest mitochondrial protein in metazoans is encoded by the male-transmitted mitogenome of the bivalve *Scrobicularia plana*

**DOI:** 10.1098/rsbl.2022.0122

**Published:** 2022-06-08

**Authors:** Mélanie Tassé, Thierry Choquette, Annie Angers, Donald T. Stewart, Eric Pante, Sophie Breton

**Affiliations:** ^1^ Département de sciences biologiques, Université de Montréal, Montréal, QC, Canada; ^2^ Department of Biology, Acadia University, Wolfville, NS, Canada; ^3^ Littoral, Environnement et Sociétés (LIENSs), UMR 7266 CNRS–La Rochelle Université, 2 rue Olympe de Gouges, 17000 La Rochelle, France

**Keywords:** mitogenomics, DNA insertion, cytochrome c oxidase subunit 2, doubly uniparental inheritance, Bivalvia

## Abstract

Cytochrome c oxidase subunit II (COX2) is one of the three mitochondrially encoded proteins of the complex IV of the respiratory chain that catalyses the reduction of oxygen to water. The *cox2* gene spans about 690 base pairs in most animal species and produces a protein composed of approximately 230 amino acids. We discovered an extreme departure from this pattern in the male-transmitted mitogenome of the bivalve *Scrobicularia plana* with doubly uniparental inheritance (DUI) of mitochondrial DNA (mtDNA), which possesses an important in-frame insertion of approximately 4.8 kb in its *cox2* gene. This feature—an enlarged male *cox2* gene—is found in many species with DUI; the COX2 protein can be up to 420 amino acids long. Through RT-PCRs, immunoassays and comparative genetics, the evolution and functionality of this insertion in *S. plana* were characterized. The in-frame insertion is conserved among individuals from different populations and bears the signature of purifying selection seemingly indicating maintenance of functionality. Its transcription and translation were confirmed: this gene produces a polypeptide of 1892 amino acids, making it the largest metazoan COX2 protein known to date. We hypothesize that these extreme modifications in the COX2 protein affect the metabolism of mitochondria containing the male-transmitted mtDNA in *Scrobicularia plana*.

## 1. Introduction

The mitochondrial DNA (mtDNA) of Metazoa is habitually depicted as a small double-stranded circular molecule of about 13–19 kb, with remarkable uniformity in gene content: it contains 37 genes, 13 of which encode protein subunits acting in oxidative phosphorylation (OXPHOS) and ATP production [1,2]. There are, however, exceptions to this typical architecture (e.g. [2,3] and see also [4–6] for newly discovered mtDNA-derived micropeptides). Notably, bivalve molluscs exhibit considerable variation in mtDNA size (less than 14 to greater than 67 kb), and supplementary, atypical protein-coding genes have also been reported [2,7–9]. The only exception to strict maternal inheritance of mtDNA in metazoans, known as doubly uniparental inheritance or DUI, is found in this group and has been reported in over 100 bivalve species [10]. DUI is characterized by two highly divergent (genetic distance greater than 40% in some species [11]) sex-linked mtDNA lineages, one that is maternally inherited (F mtDNA) and present in oocytes and somatic tissues of both sexes, and the other that is paternally inherited (M mtDNA) and present in sperm, but also sometimes in male soma [12–14].

An apparently convergent evolution of the male-specific version of the *cox2* gene (M*cox2*) represents another mitochondrial curiosity in bivalves [9]. The modifications reported in M*cox2* include long 3′ extensions or in-frame insertions [15–21]. For example, freshwater mussels (order Unionoida) have a fast-evolving 3′ extension of variable length (144–681 bp) in their M*cox2* gene, but still display a pattern of purifying selection in this additional sequence [15–17,19,21]. This extension is transcribed and translated, and its c-terminus tail has been localized at the mitochondrial surface of sperm mitochondria, leading to the hypothesis that it could serve as a tag to facilitate their sex-specific fate in bivalve embryos [17,22,23]. However, more work is needed to understand the functional importance of these modifications in the MCOX2 protein of bivalves with DUI.

The longest in-frame insertion (4827 bp) in the M*cox2* gene of a DUI bivalve has recently been reported in *Scrobicularia plana* (order Cardiida) [20], meaning that this gene could be expressed as a single polypeptide drastically larger (1892 amino acids) than the typical one (approx. 230 aa) or the enlarged one found in some male mitogenomes of other DUI species (up to 420 aa) [15–17,19–21]. However, this possibility has not been evaluated yet. Here, we used a combination of RT-PCR, immunoassays and comparative genetics to better characterize the evolution and functionality of this newly discovered M*cox2* insertion in *S. plana* and confirmed the existence of the largest mtDNA-encoded protein reported in a metazoan so far.

## 2. Material and methods

Adult specimens of *Scrobicularia plana* were collected in May 2013 from Concarneau (France; 47.8728° N, 3.9207° W), and June 2018 and 2019 from Fouras-les-Bains (France; 45.9838° N, 1.0931° W). Individuals were dissected by cutting adductor muscles and sampling a fragment of the mantle. Gonads were gently nicked with a scalpel and gametes were sampled with a P200 pipette and inspected under the microscope for sexing. Female and male gametes, mature gonads and somatic tissues were preserved in 95% ethanol or flash-frozen in liquid nitrogen and sent to the Université de Montréal on dry ice to be stored at −80°C. Total genomic DNA was extracted from gonads and somatic tissues with the Qiagen DNeasy Blood & Tissue Kit (QIAGEN 69506). RNA was extracted from gametes and somatic tissues with the *Quick*-RNA™ Miniprep kit (Zymo Research R1054) and the Nucleospin RNA for NucleoZOL kit (Macherey-Nagel 740406.50 and 740404.200). RNA was retrotranscribed into cDNA using the GoScript™ Reverse Transcription Mix (Promega A2790) and the Superscript IV 1-Step System (Invitrogen 15895891). The quality and quantity of DNA, RNA and cDNA were assessed by electrophoresis on a 1% agarose gel and/or with a BioDrop µLITE spectrophotometer. Proteins were extracted from gametes and somatic tissues by mechanical grinding and sonication in two volumes of RIPA buffer (Tris-HCL 50 mM, NaCl 150 mM, EDTA 1 mM, Triton X-100 1%, sodium deoxycholate 0.5%, sodium dodecyl sulfate [SDS] 0.1%, pH 7.6). Protein concentration was determined by Bradford protein assay kit (Sigma B6916).

**Figure 1.**
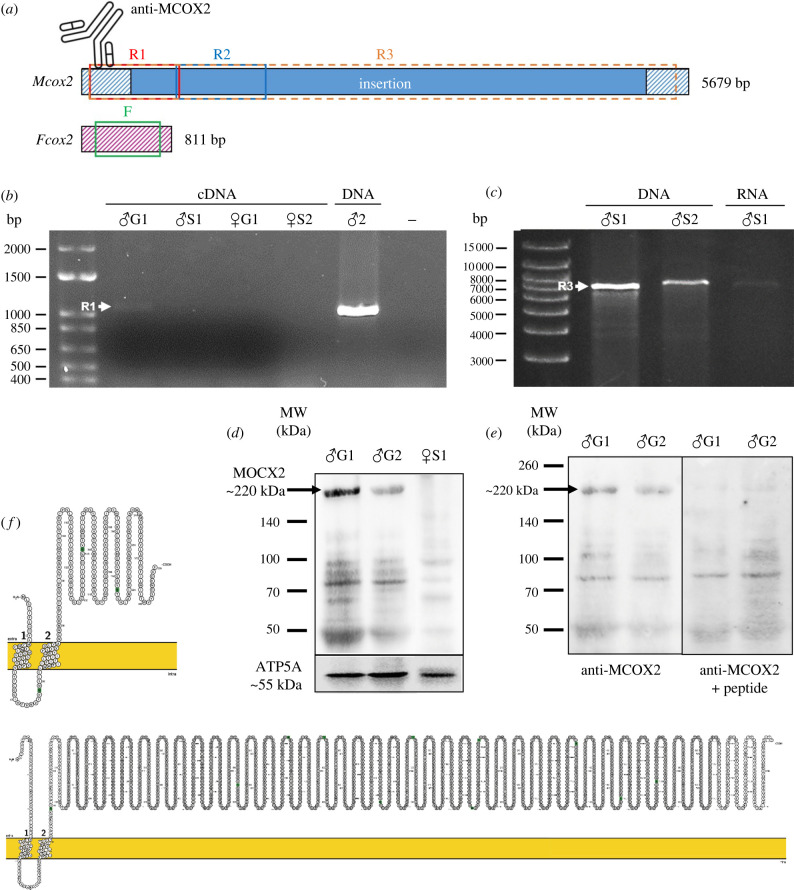
Transcription and translation of the M*cox2* insertion. (*a*) Representation of the M (blue) and F (pink) *cox2* genes and the regions targeted by PCR and RT-PCR (R1–3: red, blue and orange boxes, respectively; F*cox2*: green box). The M/F homologous region is hatched and the M*cox2* insertion is plain. The position of the epitope specific to MCOX2 is shown by an antibody. (*b*) R1 was amplified from male and female gametes (G) and somatic tissues (S) by two-step RT-PCR. (*c*) R3 was amplified from male somatic DNA by PCR and RNA by one-step RT-PCR. Amplified fragments are marked by white arrows. (*d*) Western blots detection of MCOX2 and ATP5A in sperm and female somatic tissues. (*e*) MCOX2 expression in sperm without (left) and with (right) prior incubation of antibodies with the synthetic antigenic peptide. (*f*) Predicted membrane topology model generated by Protter of FCOX2 (above) and MCOX2 (below). The inner mitochondrial membrane is represented in yellow. Amino acids from the MCOX2 insertion are in grey. Transmembrane regions are identified by 1 and 2.

Two regions of M*cox2* (R1 and R2) and one region of F*cox2* (F mtDNA) were PCR-amplified using DNA from 23 males and 18 females (figure 1*a*; electronic supplementary material, table S1 and datasets S1–S3; GenBank accession number OM928010-064). PCR products were purified with the QIAquick® Gel Extraction Kit (QIAGEN 28706) and sent to Génome Québec for Sanger sequencing in both directions. Nucleotide and amino acid sequences were aligned by MAFFT v. 7 with L-INS-I method [24]. MEGA-X was used to compute amino acid *p*-distances and rates of synonymous (*K*_S_) and non-synonymous substitutions (*K*_A_) using the Nei Gojobori method with the Jukes–Cantor correction [25]. Secondary structures of FCOX2 and MCOX2 were predicted using Protter [26]. Transmembrane alpha-helices (TMHs) were predicted using TMHMM [27], TMpred [28], Phobius [29], TOPCONS [30], InterProScan [31] and Phyre2 [32,33]. Protein three-dimensional structures were modelled using Phyre2 [33]. Function prediction was performed using BLASTp and BLASTn searches against (i) the NCBI entire non-redundant protein sequences (nr) and nucleotide sequences (nr/nt) databases, (ii) the Reference sequence (RefSeq) database, and (iii) the UniProtKB/Swiss-Prot database all with default parameters [34]. Open reading frames (ORFs) localized in the insertion with a length greater than 150 nucleotides and ATG or alternative initiation codons as start codons were found with NCBI ORF finder (https://www.ncbi.nlm.nih.gov/orffinder/, last accessed July 2021) using the invertebrate mitochondrial genetic code. Function prediction was performed for these ORFs using BLASTp against all the databases mentioned above [34]. All matches with *e*-values less than 0.001 were kept.

To check for expression, F*cox2* and two regions of M*cox2* (R1 and R3) were PCR-amplified from gamete and somatic cDNA (two-step RT-PCR) (figure 1*a*; electronic supplementary material, table S1). Male gDNA (gamete DNA) was used as a positive control, and non-treated RNA and molecular grade water were used as negative controls. The same three regions were also amplified using RNA extracted from somatic tissues with the SuperScript™ IV One-Step RT-PCR System (Invitrogen 12594025) (electronic supplementary material, table S1). Male gDNA and molecular grade water were used as negative controls. PCR and RT-PCR products were visualized on a 1% agarose gel.

To check for the translation of the full M*cox2* gene, a polyclonal antibody against the synthetic epitope CDKYKVFPHWE specific to MCOX2 was produced in rabbits and affinity-purified (MediMabs, Montréal, Canada). Protein extracts from male and female gametes and somatic tissues were supplemented with loading buffer (50 mM Tris-HCl pH 6.8, 2% SDS, 10% glycerol, 1% b-mercaptoethanol and 0.02% bromophenol blue) then heated for 5 min at 95°C before being loaded into a denaturing 4–20% polyacrylamide gel. Samples migrated for 18 h at 50 V in migration buffer (Tris 25 mM, glycine 192 mM and SDS 0.1%) and were transferred on nitrocellulose membranes at 1000 mA for 1 h 30 min in transfer buffer (glycine 1.5%, Tris base 0.3%, methanol 20%). Membranes were coloured with Ponceau S and then decoloured and blocked in a 1X phosphate-buffered saline Tween-20 (PBST; sodium phosphate monobasic (NaH_2_PO_4_) 57 mM, NaCl 154 mM, 0.05% Tween-20 and pH 8.0) with 5% BLOTTO Blocker. Membranes were incubated overnight at 4°C either with primary antibodies directed against MCOX2 (1 : 2000–1 : 20 000 in PBST) or with antibodies Anti-ATP5A (mitochondrial marker, Abcam ab14748) as positive control (1 : 2000 in PBST). Membranes were washed 3 × 15 min in PBST and then incubated for 2 h at room temperature with secondary antibodies (1 : 2000 in PBST) goat anti-rabbit-peroxidase (for membranes exposed to Anti-MCOX2 – Jackson Immuno 111-035-144) and goat anti-mouse-peroxidase (for membranes exposed to Anti-ATP5A—Jackson Immuno 115-035-003). Membranes were again washed 3 × 15 min in PBST and then washed 5 min in Tris-buffered saline (Tris-HCl 20 mM, NaCl 150 mM, pH 7.4). Finally, membranes were revealed using MBI evolution Borealis plus western blot detection (MBI BORA200ML).

## 3. Results

Figure 1*a* shows the regions of M*cox2* (R1–R3) and F*cox2* (F mtDNA) used to compute amino acid *p*-distances and rates of synonymous (*K*_S_) and non-synonymous substitutions (*K*_A_) (i.e. R1, R2 and F*cox2*), and to check for expression (i.e. R1 and R3). In the latter case, a fragment of approximately 950 bp was expected to be amplified by RT-PCR for the region R1 if the insertion is indeed transcribed (figure 1*a*). The expected signal was observed for sperm (figure 1*b*) and male somatic tissues (electronic supplementary material, figure S1A), a result consistent with the presence of M mtRNA in these tissues as has been previously noted in other species [13,35]. For the region R3 (figure 1*a*), a fragment of approximately 5.6 kb was expected to be amplified (with the insertion fully transcribed). PCR amplification on sperm cDNA failed to detect such a fragment (electronic supplementary material, figure S1B), but one-step RT-PCR on male somatic RNA samples showed a faint band of approximately 7 kb (figure 1*c*). It is possible that very low levels of mitochondrial RNA and retrotranscribed mtRNA were present in sperm (this added to the difficulty of retrotranscribing very long RNA molecules). The approximately 1.5 kb difference between the expected length of R3 and the visualized length might be due to insertions/deletions (indels) found within the M*cox2* insertion (see below). Strong bands of expected size were obtained for F*cox2* (electronic supplementary material, figure S1C, D).

In support to RT-PCR results, western blots revealed a band of approximately 220 kDa only in sperm (figure 1*d*). This is slightly heavier than the predicted mass of the protein (204 kDa) (ExPASy server; [36]) but could be attributed to the presence of indels as observed in our sequences, and/or to the presence of TMHs or post-translational modifications such as glycosylation or ubiquitination [37]. To verify the specificity of the anti-MCOX2 antibody, the synthetic antigenic peptide was added to the primary antibody solution at a 10 X concentration before the incubation to competitively chelate every antigenic site of the primary antibody and to show the attenuation of the specific MCOX2 band (figure 1*e*). This result confirmed that the in-frame insertion in the M*cox2* gene is translated.

FCOX2 and MCOX2 amino acid sequences differed by 50% (table 1). Genetic distances were low within sexes (less than 2.6%), the lowest being among FCOX2 and the highest among MCOX2 insertions (table 1). *K*_A_/*K*_S_ ratios were all indicative of purifying selection (less than 1 for all regions; table 2).

**Table 1.** Intraspecific amino acid divergences between and within FCOX2 and MCOX2. *Note*. The pairwise deletion option computes a distance for each pair of sequences and ignores gaps involved in the comparison. The complete deletion option does not compute any sites with gaps or missing information in the comparison. R1homo refers to the homologous portion of R1 with F*cox2*; R1ins refers to the insertion portion of R1.

			pairwise deletion		complete deletion	
	sample size (*n*)	sequence length (aa)	mean number of amino acid differences (aa)	*p*-distance	mean number of amino acid differences (aa)	*p*-distance
FCOX2 vs MCOX2	36	238	34.940 ± 5.531	50.390% ± 6.123%	32.441 ± 4.013	50.689% ± 5.836%
F*cox2*	18	176	0.737 ± 0.266	0.419% ± 0.152%	0.737 ± 0.272	0.419% ± 0.159%
R1homo	17	90	1.471 ± 0.464	1.634% ± 0.535%	1.471 ± 0.486	1.634% ± 0.532%
R1ins	17	169	4.029 ± 0.851	2.537% ± 0.526%	3.081 ± 0.752	2.233% ± 0.540%
R2	21	278	6.295 ± 1.081	2.491% ± 0.452%	5.229 ± 1.065	2.551% ± 0.530%

**Table 2.** Synonymous (*K*_S_) and non-synonymous *(K*_A_) substitution rates of F*cox2* and M*cox2* gene regions. *Note*. R1homo and R1ins refer to the homologous portion of R1 with F*cox2* and to the insertion portion of R1, respectively.

	*K*_A_	*K*_S_	K_A_/K_S_
F*cox2*	0.001829 ± 0.000676	0.020877 ± 0.005271	0.087608 ± 0.054499
R1homo	0.007939 ± 0.002405	0.022851 ± 0.009657	0.347425 ± 0.252072
R1ins	0.012346 ± 0.002857	0.040441 ± 0.009866	0.305284 ± 0.145123
R2	0.011443 ± 0.002030	0.042749 ± 0.007393	0.267679 ± 0.093779

In metazoans, the N-terminal domain of the cytochrome c oxidase subunit II (COX2) protein typically contains two TMHs, which are followed by a ‘heme-patch’ region and two Cu_a_-binding centres [20,38]. The insertion in the protein MCOX2 is localized between the ‘heme-patch’ region and the first Cu_a_-binding centre [20]. Secondary structure and TMH predictions suggested that MCOX2 possesses the two conserved TMH located near the N-terminus end (figure 1*f*; electronic supplementary material, table S2). The three-dimensional model of MCOX2 predicted by Phyre2 had a high precision value (99.6%) but a small coverage (6%) including only a part of the F/M homologous C-terminal region (electronic supplementary material, figure S2).

BLASTn searches using only the M*cox2* insertion revealed hits with part of European starfish *Asterias rubens* chromosome 14 (3% coverage, approx. 74% similarity, *e*-value 2 × 10^−12^) and with a translation initiation factor IF-2 in sorghum *Sorghum bicolor* (2% coverage, approx. 69% similarity, *e*-value 7 × 10^−4^) (electronic supplementary material, table S3). BLASTp searches using only the MCOX2 insertion revealed hits (most of bacterial origin) with a small query coverage (less than or equal to 5%) and percentage identity of less than 52% (electronic supplementary material, table S3). A conserved domain was identified, i.e. PHA03247 large tegument protein UL36 (*e*-value 3.06 × 10^−4^). Also, out of the 27 ORFs greater than 150 nt found in the insertion, there were three with significant similarity to at least one sequence (electronic supplementary material, table S3). One ORF, covering almost the entire insertion, had identical hits to the one from the complete insertion. Two other ORFs with a length of approximately 100 aa and localized on the reverse strand returned hits referring to a myosin light chain kinase (61% coverage, approx. 48% similarity, *e*-value 5 × 10^−4^) and a phosphoesterase (53% coverage, approx. 45% similarity, *e*-value 7 × 10^−6^) (electronic supplementary material, table S3).

## 4. Discussion

Herein, we report the longest mitochondrially encoded protein in the animal kingdom (1892 amino acids). The important in-frame insertion of approximately 4.8 kb previously reported in the male *cox2* gene of the DUI bivalve *Scrobicularia plana* [20] results in a significant enlargement of the protein: (i) the insertion does not contain any premature stop codon, it is conserved among individuals from different populations (over 300 km apart) and it bears the signature of purifying selection, which can be interpreted as a way to maintain a functional protein and (ii) the insertion is transcribed and a protein of approximately 220 kDa was detected in sperm by western blot analysis, which corresponds to the expected size of the MCOX2 peptide with the insertion. We believe that our observations are not the result of nuclear mtDNA segments (NUMTs; [39]) because (i) the complete male mitogenome of *S. plana* has been assembled with MITObim [20], a program expected to perform well even for species with high number of NUMTs [40], (ii) NUMTs greater than 4 kb are usually rare in animal genomes, and they are mostly non-functional pseudo-genes and rarely expressed [41–43], (iii) we were able to amplify large portions of M*cox2* both at the DNA and RNA levels, and (iv) the M*cox2* gene cannot be completely translated using the universal genetic code (in particular, there is no possible ORF > 50 aa using the universal genetic code for the region containing the epitope CDKYKVFPHWE specific to MCOX2 used to generate our antibody).

To our knowledge, the only other animal species with an important in-frame insertion (greater than 3 kb) in its *cox2* gene is the hymenopteran *Campsomeris* [44]. However, contrary to the situation in *S. plana*, *cox2* in *Campsomeris* is split into two genes encoding two complementary polypeptides, COX2A and COX2B, which apparently form a heterodimer [44]. This insertion between *cox2a* and *cox2b* possesses several antiparallel overlapping ORFs, one of which encodes a nuclease potentially involved in *cox2* fission [44]. Such ORFs were not found in *S. plana*, but a conserved domain was identified in the insertion, i.e. PHA03247 large tegument protein UL36, which is conserved among herpesviruses and plays many roles in viral replication [45]. Interestingly, this result is in line with a previous hypothesis that viral selfish elements may have colonized the male mitogenome in bivalves promoting its segregation into primordial germ cells and allowing its transmission to next generations [8,46]. It is also worth mentioning that herpesviruses were reported in many bivalve species [47,48].

As suggested by Curole & Kocher [16], it is tempting to speculate that extensions and insertions in M*cox2* of bivalves with DUI are a result of the new male-specific selective environment. According to previous [15–17,20] and present analyses, the two TMHs, the ‘heme-patch’ region and the two Cu_a_-binding centres are conserved in MCOX2 of DUI bivalves, suggesting that its role in OXPHOS is conserved. In the case of *S. plana*, secondary structure and TMH predictions suggested that the insertion is located in the mitochondrial intramembrane space, but additional putative TMHs predicted by some programs (see electronic supplementary material, table S2) could allow the protein to be exposed at the mitochondrial surface. This was reported for the MCOX2 C-terminal extension of freshwater mussels, and it is the reason why the extension has been hypothesized to act as a tag for paternal mitochondria [17,23]. However, this remains to be demonstrated.

We suggest that the apparently convergent evolution of the M*cox2* gene might play a part in the reorganization of sperm energy metabolism in bivalves with DUI [49–51]. Specifically, eggs and soma of DUI species, usually homoplasmic for the F mtDNA, express a common mitochondrial ‘F-phenotype’, whereas sperm and their M-type mitochondria express an ‘M-phenotype’, which is characterized by low OXPHOS rates and an almost null spare capacity of the cytochrome c oxidase complex [49,51]. This sperm-specific, potentially male-inducing mitochondrial phenotype in DUI species [52] could be due, at least in part, to modifications in the M*cox2* gene. Physiological comparisons of several DUI taxa with and without a functional M*cox2* extension or insertion will provide an opportunity to test this hypothesis. Such studies will also help to better understand if these important modifications in the M*cox2* gene of DUI species may represent a male-specific energetic adaptation to enhance fertilization success.

## Data Availability

All data are available as electronic supplementary material [53].
